# Use of elder donors for cadaveric single kidney transplantation: a new evolution or an inacceptable risk?

**DOI:** 10.1186/1471-2318-11-S1-A34

**Published:** 2011-08-24

**Authors:** F Melandro, Q Lai, F Nudo, G Spoletini, GB Levi Sandri, L Poli, R Pretagostini, PB Berloco

**Affiliations:** 1 Department of General Surgery and Organ Transplantation, Sapienza University, Rome, Italy

## Background

Organ shortage and long waiting times represent relevant issues in modern kidney transplantation [1]. Expansion of the donor pool using Extended Criteria Donors (ECD) represents a way to partially resolve these limits. ECDs are defined by UNOS as ≥ 60-year aged donors or 50-59-year aged donors with at least 2 of 3 risk factors (pre-procurement serum creatinine >1.4 mg/dl, cerebrovascular accident and history of hypertension) [2]. However, use of ECD seems to be related to worse results in terms of graft function and survival [3]. Moreover, no data exist with regard to comparison between over-60 and 50-59-year aged donors. The aim of this study is to analyze the cohort of ECD transplants performed in our Department, evaluating the role of donor age on results.

## Materials and methods

From January 2004 to May 2009, 95 single kidney transplantations using ECDs were performed. The entire cohort was stratified in 2 groups: Group A (50-59 years, n=26) and Group B (≥ 60 years, n=69). Donor, recipient and transplant characteristics were compared using the chi-squared and the Mann-Whitney test. Patient and graft survival were analyzed by the Kaplan-Meier method and compared using the log-rank test.

## Results

**Figure 1:**
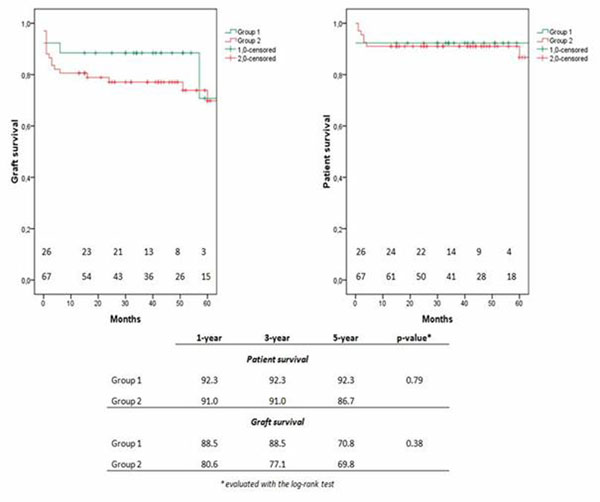
Graft and patient survivals in the 2 groups

Group A presented younger donors (55 *vs* 67 years) and recipients (53 *vs* 58 years), a higher number of donors with previous history of hypertension (92% *vs* 43%) and higher pre-harvesting creatinine values (1.2 *vs* 0.9 mg/dL). Post-transplant graft function did not present statistical differences. Five-year patient and graft survivals results were similar (Fig. 1).

## Conclusions

Use of ECD seems to be safe, even using very elderly donors. In our experience, biopsy-driven seection is exclusively performed in over-60 donors. Starting from this consideration, we could speculate that the use of biopsy in over-60 donors allows “bad donors” to be excluded obtaining similar survival rates with respect to younger donors. Systematic use of biopsy in 50-59-year donors with risk factors could further improve outcomes.
